# Cryptococcus: Emerging host risk factors for infection

**DOI:** 10.1371/journal.ppat.1013602

**Published:** 2025-11-05

**Authors:** Alejandro L. Antonia, J. Andrew Alspaugh

**Affiliations:** 1 Department of Medicine, Duke University School of Medicine, Durham, North Carolina, United States of America; 2 Department of Molecular Genetics and Microbiology, Duke University School of Medicine, Durham, North Carolina, United States of America; 3 Department of Cell Biology, Duke University School of Medicine, Durham, North Carolina, United States of America; University of Maryland, Baltimore, UNITED STATES OF AMERICA

## Introduction

**Fig 1:**
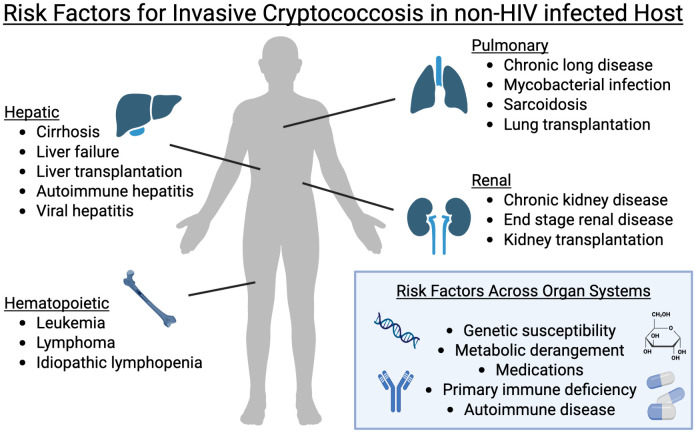
Risk factors for invasive cryptococcosis in non-HIV-infected hosts. Multiple retrospective studies have identified hepatic, renal, pulmonary, and hematopoietic dysfunction as associated with increased risk of *Cryptococcus* infection. Beyond specific organ dysfunction, there is growing appreciation for the role of host genetics, metabolic derangements, iatrogenic immunosuppression, and dysregulated immune response increasing cryptococcosis risk. Created in BioRender. Antonia, A. (2025) https://BioRender.com/rtkbe07.

Invasive cryptococcal disease is a feared complication for patients with immunodeficiencies. Historically a rare cause of human disease primarily in patients with hematologic malignancies, *Cryptococcus neoformans* became a significant cause of life-threatening opportunistic infections during the HIV pandemic [1]. *C. neoformans* typically establishes primary infection in the lungs, and it can disseminate to other sites with devastating complications, such as cryptococcal meningitis [2]. Globally, invasive *Cryptococcus* infections result in an estimated 152,000 cases of meningitis and 112,000 deaths annually, primarily in HIV positive individuals [3]. However, there is growing appreciation for invasive *Cryptococcus* spp. infection among non-HIV infected hosts (studies with published statistically significant associations between *Cryptococcus spp.* infection and comorbid conditions are summarized in Table 1 and Fig 1) [4–21]. These demonstrate that non-HIV infected hosts often have worse outcomes including higher mortality. However, several features of these studies, including varied case definitions of cryptococcosis often relying on hospital billing codes rather than direct microbiologic evidence and heterogenous study designs relying on retrospective analyses, limit the ability to make broad estimates of absolute or relative risk of invasive cryptococcosis in HIV negative patients.

**Table 1:** Summary of risk factors for invasive cryptococcosis in non-HIV hosts. Studies assessing risk factors for *Cryptococcus neoformans* with at least >40% of study population being HIV negative. Statistically significant positive associations with comorbid disease states are denoted in blue.

Study Characteristics (including ≥ 40% HIV negative population)							Significant Association Reported with Comorbid Disease						
Study	Design	Dates	Location	Case Definition	Population Size		Liver	Kidney	Lung	Malignancy	Autoimmune	Diabetes	Sarcoid
[4]	Retrospective Case Study	1990-1996	United States	Positive culture, histology, or serum/CSF antigen with associated syndrome	n = 306 (HIV = 0)								
[5]	Retrospective Cohort	1996-2009	United States	ICD9 code: cryptococcosis and cryptococcal meningitis with confirmed infection	n = 207 (HIV n = 86)								
[6]	Retrospective Cohort	1996-2010	United States	Positive blood/body fluid/tissue/sputum culture; or serum/CSF antigen; with compatible syndrome	n = 302 (HIV n = 108)								
[7]	Retrospective Cohort	1997-2009	United States	ICD9 code: Cryptococcal meningitis; excluding ICD9 code for HIV	n = 6689 (HIV = 0)								
[8]	Retrospective Cohort	2001-2010	France	ICD10 code: cryptococcosis	n = 1859 (HIV n = 656)								
[9]	Retrospective Case Control	2002-2010	Taiwan	ICD9 code: cryptococcosis; confirmation with positive CSF or blood culture	n = 158 (HIV n = 42)								
[10]	Retrospective Cohort	2002-2017	United States	Positive culture; or serum/CSF antigen; or ICD9 code: cryptococcus	n = 304 (HIIV n = 105)								
[11]	Retrospective Cohort	2004-2012	United States	ICD9 code: cryptococcosis and cryptococcal meningitis	n = 3628 (HIV n = 2091)								
[12]	Retrospective Case Series	2005-2017	United States	ICD9/10 code: cryptococcosis and cryptococcal meningitis with confirmed infection	n = 114 (HIV n = 23)								
[13]	Retrospective Observational	2010-2017	Japan	Positive serum/CSF antigen	n = 533 (HIV n = 64)								
[14]	Retrospective Case Control	2016-2023	China	Diagnosis: cryptococcosis, cryptococcal meningoencephalitis, or disseminated cryptococcosis	n = 106 (HIV n = 0)								

Nonetheless, there are co-morbid diseases that have been associated with the risk of cryptococcosis in multiple independent studies (Table 1). Increasing reports of invasive fungal disease in individuals without traditional risk factors emphasizes the need to understand how emerging host conditions, which are not overly immunocompromising, such as chronic metabolic disorders, influence outcomes of host-pathogen interactions. Interestingly, pathogen-specific risk factors are also potentially changing. For example, infections due to other related *Cryptococcus* spp. such as *C. gattii* have a propensity to cause life-threatening disease in apparently immunologically normal individuals (see Box 1 for further discussion of pathogen diversity as a risk for disease). This review highlights how the changing human epidemiology of invasive cryptococcosis informs our understanding of host-specific risk for infections and presents opportunities to better understand the immune response to fungal challenges.

## Hematologic malignancy

Hematologic malignancy represents a broad class of diseases with diverse etiologies and treatments, where risk of opportunistic infections can arise from either the malignancy or treatment. Epidemiologic studies of cryptococcal infections in non-HIV and non-transplant hosts consistently identify hematologic malignancies as risk factors for invasive cryptococcal disease. However, these have been underpowered to identify specific syndromes associated with increased risk of invasive cryptococcosis. Additionally, guidelines from the American Society of Clinical Oncology and Infectious Diseases Society of America recommend azole-based primary anti-fungal prophylaxis for patients at risk for prolonged neutropenia. These recommendations have resulted in a reduction in the incidence of invasive fungal infections including cryptococcosis [22]. Within these limitations, a literature review from 1970 to 2014 found a disproportionate enrichment of cryptococcosis among patients with non-Hodgkins lymphoma and chronic lymphocytic leukemia [23], perhaps due to underlying immune cell dysfunction or a consequence of cytotoxic chemotherapy. Future prospective studies are needed to address whether distinct hematologic malignancies associated with perturbations in protective anti-cryptococcal immune responses are associated with clinical infection.

In addition to impaired immunity related to primary disease, the treatments for hematologic malignancies frequently lead to iatrogenic immune deficiency. In the extreme, hematopoietic stem cell transplantation results in a profound state of immune deficiency. In addition, high-dose and prolonged corticosteroid use are associated with invasive cryptococcosis [24]. Further, new therapies, including small molecules, monoclonal antibodies, and antibody-drug conjugates, are being developed that can target specific dysregulated immune signaling pathways in these cancers. Additionally, chimeric antigen receptor T-cell (CAR T-cell) therapy is increasingly used to target hematologic malignancies that are recalcitrant to standard therapies. Although no specific studies have been published to date regarding associations between this therapy and cryptococcosis, investigators are exploring using CAR T-cells directed against cryptococcal capsule antigens as a novel way to treat this life-threatening infection [25]. With the advent of these targeted therapies, a shift in our framework for thinking about general risk of infection is warranted. While perhaps not as globally immunosuppressive as general cytotoxic therapies, there are well-described but nuanced associations between specific immunomodulators and narrower subsets of infections. For example, the Bruton’s Tyrosine Kinase Inhibitor, ibrutinib, has been reported to be associated with several invasive fungal infections including aspergillosis and cryptococcosis [26]. Other small molecule kinase inhibitors such as ruxolitinib (a Janus-activated Kinase inhibitor) have been associated with increased incidence of cryptococcosis. Additionally, sorafenib (MAP Kinase inhibitor) and fostamatinib (Syk inhibitor) have been associated with infections due to other fungal pathogens [27,28]. As use of novel immune-based therapies increases, it will be important to proactively identify emerging associations between specific immune-modulating agents and invasive fungal infections to develop opportunities for prophylaxis, early diagnosis, and rapid treatment.

## Solid organ transplantation

Solid organ transplant patients are maintained on immunosuppressive medications to reduce the risk of organ rejection. This immunosuppression also leads to an increased risk of infection. Among solid organ transplant recipients, cryptococcosis is the third most common invasive fungal infection [29] and is associated with increased mortality compared to HIV-infected populations [5]. Estimates of annual incidence of cryptococcal infection after solid organ transplant range from 0.2% to 5.0% [29–33]. Infection typically occurs 1–2 years after transplantation, consistent with new environmental exposure or reactivation rather than donor-derived infection [29,31,32]. The contribution of specific organ transplant type to the risk for cryptococcal disease remains unclear, as different studies have reported varied associations with incidence of cryptococcosis with kidney [33], heart [30], small bowel [30], and lung transplant [32]. These observations of widely variable infections likely reflect heterogeneity in immunosuppressive regimens and complications of failed transplant. For example, the net-state of immunosuppression required to avoid immune-mediated organ rejection contributes to overall risk of infection. On the other hand, specific therapies such as maintenance immunosuppression with calcineurin inhibitors can be associated with decreased mortality from cryptococcus infection in part due to anti-fungal activity of these compounds in addition to modulation of inflammatory response [34], emphasizing the challenges and opportunities from designing therapeutics in eukaryotic pathogens. Additionally, observations of increased mortality from cryptococcal infection in the setting of liver and renal transplantation may also reflect dysfunction of the underlying organ system (see further discussion below) [30,34]. As the total population of transplant patients continues to increase [35], combining increased resolution of clinical data with detailed mechanistic studies will be paramount to understand who is at risk for infection and inform novel therapeutic and prevention strategies.

## Primary immune deficiency and host genetic susceptibility

A diverse set of primary immune deficiencies places patients at risk for more frequent and severe infections including invasive fungal infections [36]. *C. neoformans* infection has been described in disorders that affect both the innate and adaptive immune system such as severe combined immunodeficiency (SCID) [37], hyper IgE syndrome [38], hyper IgM syndrome [39], and idiopathic CD4 lymphopenia [40]. Additionally, the observation of invasive cryptococcal disease in patients with apparently normal immune systems spurred investigations into potential novel mechanisms of immune deficiencies.

Advances in our ability to immunophenotype patients without known risk factors for invasive fungal infections has demonstrated differences in cytokine production, baseline T-cell populations, and auto-antibodies reactive against key immune signaling pathways [41]. For example, auto-antibodies to cytokines such as interferon-γ and granulocyte-macrophage colony stimulating factor (GM-CSF) have been observed in patients with invasive cryptococcosis and no other identifiable immune deficiency [21,42]. Retrospective observational [43] and cohort studies have reported anti-GM-CSF antibodies in up to 8.1% of HIV-noninfected patients with cryptococcal meningitis. Further, anti-GM-CSF antibodies are associated with differences in *Cryptococcus* species, disease phenotype, and mortality [42]. New case reports of anti-GM-CSF antibodies in the context of other opportunistic pathogens emphasize a continued role for *C. neoformans* as a model system for interrogating host immunity [44].

Advances in human genetics have also enabled discoveries into previously unrecognized host susceptibility to invasive cryptococcal disease [45]. Due to the limited sample size of invasive cryptococcal disease in non-HIV hosts, no unbiased genome wide association studies in this population have been conducted. Nonetheless, hypothesis-driven studies have identified variants in genes with known protective cryptococcal immune function to be associated with poor outcomes in human disease. For example, risk of invasive cryptococcal disease has been associated with variants involved in both indirect pathogen sensing through FCγ receptors [46,47] or direct pathogen sensing through pattern recognition receptors including mannose-binding lectin 2 [48], dectin-2 [49], and toll-like receptors [50]. The only genome-wide association study for cryptococcosis was conducted in HIV-infected patients and identified the single-nucleotide polymorphism (SNP) rs1999713 upstream of the gene encoding the cytokine macrophage colony-stimulating factor (M-CSF). M-CSF, a structurally related cytokine to GM-CSF but with distinct immune signaling, was also differentially expressed in macrophages after stimulation with *C. neoformans,* and exogenous M-CSF was able to enhance macrophage phagocytosis and killing of *C. neoformans* [51]. As in the case of the FCγ receptor polymorphisms, this discovery in a different patient population represents an opportunity to test novel hypotheses regarding risk of cryptococcal disease in non-HIV-infected hosts.

## Metabolic syndrome and hepatic dysfunction

Three diseases associated with aging and metabolic syndrome are consistently observed to have increased risk for invasive cryptococcosis in non-HIV-infected hosts: hepatic dysfunction, renal disease, and diabetes mellitus (Table 1). Additionally, despite commonalities in the mechanism of acquiring these diseases with chronic physiologic changes from metabolic syndrome, they likely have unique mechanisms of immune deficiency leading to increased susceptibility to *Cryptococcus* infection.

### Hepatic dysfunction and cirrhosis

Liver disease is reported as a common organ dysfunction syndrome leading to invasive *Cryptococcus* infection (Table 1) including atypical presentations such as peritonitis [52,53] and pericarditis [54]. Further, a retrospective case-control study of 188 cases of invasive cryptococcosis identified decompensated liver cirrhosis to convey the second highest risk of cryptococcal disease after advanced HIV infection (adjusted odds ratio of 8.5) [9]. Additionally, *Cryptococcus* infection in patients with liver disease has repeatedly been associated with significantly higher mortality compared to similar infections in other patient populations [55,56]. While the mechanism for immune deficiency leading to increased cryptococcal disease specifically has not been identified, cirrhosis is increasingly recognized to lead to broadly dysregulated immune responses.

### Diabetes mellitus

While a cryptococcal-specific mechanism of susceptibility for diabetes mellitus has not been identified, this disease is recognized to broadly lead to increased susceptibility to infectious diseases [57]. It has been demonstrated to increase risk of infection with *Cryptococcus* spp. in whole population level studies (Table 1) and in focused studies specifically examining patients with diabetes [58]. As a disorder of metabolic function, diabetes mellitus as a risk factor for cryptococcal disease highlights the potential role of nutritional immunity in effective fungal control. Under host-derived stress conditions, *Cryptococcus neoformans* upregulates numerous genes involved in nutritional regulation including of sugars, metals, and amino acids [59]. In other fungal diseases such as invasive candidiasis, elevated blood glucose has been linked to increased susceptibility to infection by increasing available energy sources for pathogens and altered host immune signaling [60]. While in the case of invasive mucormycosis, secondary metabolic changes to available iron concentrations in the context of diabetic ketoacidosis also directly contribute to pathogenesis [61]. Interestingly, recent studies of cryptococcosis in mice have shown that ketogenic diets may favor the clearance of infection in combination with antifungal therapy. These and subsequent studies will further enrich our understanding of the role of metabolism on host and microbial factors during infection [62].

### Renal dysfunction

While renal homeostasis has roles in modulating local and systemic immune responses [63], there has yet to be a clearly defined mechanism that explains the increased risk of invasive cryptococcal disease with renal dysfunction (Table 1). However, patients with renal disease are more likely to die from cryptococcal infection (OR of 2.45 to 3.5) [8,13]. Further, the association between renal disease and invasive cryptococcosis has been replicated in multiple follow-up studies with specific patient populations including renal transplant patients at increased risk of cryptococcal disease acquisition, dissemination, and mortality [33,34]; and HIV-uninfected general populations without transplantation in which chronic kidney disease was associated with an increased risk of disseminated cryptococcosis and mortality [64].

## Autoimmune disease and sarcoidosis

Autoimmune diseases have varied reports of increased relative prevalence of cryptococcal infection (depending on the underlying disease ranging from 5.4 times more likely up to 17 times more likely than the general population) likely reflecting the heterogenous nature of these diseases [7]. They include systemic lupus erythematosus, rheumatoid arthritis, multiple sclerosis, and sarcoidosis. The increased susceptibility to *Cryptococcus* infection certainly reflects a combination of underlying immune dysregulation, immunomodulatory treatments, and associated structural lung disease.

This combination of risk factors is illustrated by the example of sarcoidosis. In a retrospective review of all cases of cryptococcosis in France, significantly more non-HIV-infected patients were found to have sarcoidosis [65]. Of 18 patients with both sarcoidosis and cryptococcosis, 12 were receiving immunosuppressive medications including corticosteroids placing them at high risk of infection, whereas 4 had not received prior immunosuppressive medications. This demonstrates that underlying immune dysfunction or structural lung disease are risk factors independent of iatrogenic immunosuppression.

While management of sarcoidosis typically involves non-specific immunosuppression, recent reports from targeted therapies for multiple sclerosis (MS) demonstrate how novel immunomodulatory medications might unexpectedly increase risk. For example, the sphingosine-1-phosphate (S1) analog FTY720 (fingolimod) decreases central nervous system inflammation by preventing lymphocyte exit from peripheral lymphoid tissue, and by directly modulating immune signaling of effector cells [66]. Sixty patients receiving this therapy from 2006 to 2020 developed cryptococcal infection [67]. A review of 25 clinical cases identified lymphopenia as a risk factor for invasive cryptococcosis among patients on fingolimod [68], and murine studies demonstrate that fingolimod impairs macrophage function via an S1P receptor-dependent mechanism, which leads to increased reactivation of *Cryptococcus* infection from pulmonary granulomas [69].

Together, these studies highlight the interaction between underlying immune dysfunction, structural disease, and iatrogenic immune modulation that can contribute to *C. neoformans* infection risk in increasingly subtle subsets of patients with autoimmune disease.

## Conclusion

Invasive cryptococcal infection in non-HIV-infected hosts is well described and associated with many adverse outcomes. As more reports become available describing cryptococcosis in this patient population, the details of under-recognized risks for fungal infections are coming into focus. It is also clear that this picture is expanding in the context of increasing transplantation, novel immunomodulatory medications, and the epidemic of chronic metabolic disorders associated with aging. With this expanding population, there will be new opportunities to better understand mechanisms by which *Cryptococcus* spp. cause invasive disease and therefore predict who is at risk in order to develop novel, safer modalities for treatment.

**Box 1. Pathogen variation as a risk factor for invasive cryptococcosis.**

Fungal genetic heterogeneity contributes to risk and variable presentation of invasive cryptococcosis. There are increasing reports of infection with the related species *C. deneoformans* and *C. gattii;* the latter of which is a more frequent cause of clinically recognized infections in non-HIV-infected populations [18]. Additionally, genome-wide association studies of *Cryptococcus* isolates have identified genetic variants associated with differences in fungal burden, immune recognition, and immune signaling [70,71]). While discussion of fungal genetic diversity is beyond the scope of this review, as more genetic data becomes available from clinical isolates, it is likely that pathogen genetic variation will be a key variable to explain risk and outcome of cryptococcal infection.

**Box 2. Increasing complexity of host immunomodulating therapies.**

Over the last several decades, there has been an increasing number of patients treated with rapidly diversifying types of immune-modulating therapies. Historically, these have been broadly immunosuppressive, such as corticosteroids and cytotoxic chemotherapy which were both individually recognized as risk for invasive fungal diseases. More recently, modulation of specific immune signaling pathways, through small molecules and monoclonal antibodies, have been less globally immunosuppressive but can still have increased susceptibility to specific subsets of pathogens depending on their unique mechanism of action. Several of these therapies, notably monoclonal antibodies that target immune checkpoints, can theoretically augment pathogen-specific immune responses. Additionally, patients are increasingly being exposed to novel combinations of these immunosuppressives. Going forward, it will be important for clinicians to be aware of new at-risk populations that emerge in this context, and when a new association arises, tackle the opportunity to learn more about invasive fungal infections.
